# Associated Clinical and Laboratory Markers of Donor on Allograft Function After Heart Transplant

**DOI:** 10.5935/1678-9741.20160025

**Published:** 2016

**Authors:** Renato Braulio, Marcelo Dias Sanches, Antonio Lúcio Teixeira Junior, Paulo Henrique Nogueira Costa, Maria da Consolação Vieira Moreira, Monaliza Angela Rocha, Silvio Amadeu de Andrade, Cláudio Léo Gelape

**Affiliations:** 1 Federal University of Minas Gerais (UFMG), Belo Horizonte, MG, Brazil.

**Keywords:** Heart Transplantation, Tissue Donors, Biomarkers, Norepinephrine, Primary Graft Dysfunction

## Abstract

**Introduction:**

Primary graft dysfunction is a major cause of mortality after heart transplantation.

**Objective:**

To evaluate correlations between donor-related clinical/biochemical markers and the occurrence of primary graft dysfunction/clinical outcomes of recipients within 30 days of transplant.

**Methods:**

The prospective study involved 43 donor/recipient pairs. Data collected from donors included demographic and echocardiographic information, noradrenaline administration rates and concentrations of soluble tumor necrosis factor receptors (sTNFR1 and sTNFR2), interleukins (IL-6 and IL-10), monocyte chemoattractant protein-1, C-reactive protein and cardiac troponin I. Data collected from recipients included operating, cardiopulmonary bypass, intensive care unit and hospitalization times, inotrope administration and left/right ventricular function through echocardiography.

**Results:**

Recipients who developed moderate/severe left ventricular dysfunction had received organs from significantly older donors (*P* =0.020). Recipients from donors who required moderate/high doses of noradrenaline (>0.23 µg/kg/min) around harvesting time exhibited lower post-transplant ventricular ejection fractions (*P* =0.002) and required longer CPB times (*P* =0.039). Significantly higher concentrations of sTNFR1 (*P* =0.014) and sTNFR2 (*P* =0.030) in donors were associated with reduced intensive care unit times (≤5 days) in recipients, while higher donor IL-6 (*P* =0.029) and IL-10 (*P* =0.037) levels were correlated with reduced hospitalization times (≤25 days) in recipients. Recipients who required moderate/high levels of noradrenaline for weaning off cardiopulmonary bypass were associated with lower donor concentrations of sTNFR2 (*P* =0.028) and IL-6 (*P* =0.001).

**Conclusion:**

High levels of sTNFR1, sTNFR2, IL-6 and IL-10 in donors were associated with enhanced evolution in recipients. Allografts from older donors, or from those treated with noradrenaline doses >0.23 µg/kg/min, were more frequently affected by primary graft dysfunction within 30 days of surgery.

**Table:** 

Abbreviations, acronyms & symbols				
BNP	= B-type natriuretic peptide		PGD	= Primary graft dysfunction
CPB	= Cardiopulmonary bypass		PVR	= Pulmonary vascular resistance
CRP	= Cardiopulmonary bypass		sTNFR1	= Soluble tumor necrosis factor receptors 1
CRP	= Cardiopulmonary bypass		sTNFR2	= Soluble tumor necrosis factor receptors 2
FiO_2_	= Fraction of inspired oxygen		TBI	= Traumatic brain injury
IL	= Interleukins		TNIU	= Troponin I ultra
IL	= Intensive care unit		VEF	= Ventricular ejection fraction
IL	= Monocyte chemoattractant protein-1			

## INTRODUCTION

Heart failure is one of the major causes of hospitalization worldwide, particularly for individuals aged 65 years and above. In cases where the condition becomes clinically refractory, heart transplantation appears to be the best therapy with satisfactory outcomes having been established during the last decades^[1]^. However, the number of heart transplants performed annually has leveled off over the last 20 years mainly because of the chronic shortage of viable donated organs. This situation has led transplant centers to accept hearts from marginal donors in an effort to expand the pool of organs available to severely ill patients on the priority waiting list, and the strategy has yielded satisfactory results^[2]^.

The principal criteria for rejecting heart donors, apart from age and other harvesting-related issues, are systolic dysfunction and myocardial hypertrophy on echocardiography since they represent key risk factors for post-transplant outcome^[3]^. However, appropriate clinical management of the potential organ donor can often alleviate such problems, thereby increasing the number of eligible donors and optimizing organ function for the purposes of transplantation^[4]^. Nevertheless, despite careful assessment and treatment of potential donors, primary graft dysfunction (PGD) still occurs in approximately 20% of cases and is one of the major causes of mortality after heart transplantation even when quality donors are involved^[5]^.

Donor-specific clinical or biochemical markers that can be used to predict the quality of a cardiac graft have yet to be firmly established. However, some evidence suggests that increased levels of procalcitonin, cardiac troponin T (cTnT) and B-type natriuretic peptide (BNP) may be independent predictors of PGD^[6,7]^. In addition, the inflammatory status of the potential donor, as evaluated from cytokine levels, appears to influence the quality of the allograft, while doses of inotropic agents (catecholamines such as noradrenaline, adrenaline, dopamine or dobutamine) administered at the time of harvesting represent an important risk factor for PGD^[8-12]^.

Most of the practices and guidelines relating to the management of donor organs follow predefined physiological and biochemical parameters in order to improve graft function and patient survival, while more novel approaches include non-conventional variables such as the measurement of plasma cytokines to determine the inflammatory status of the donor^[11]^. However, the quality of the allograft may depend on the interaction between pro- and antiinflammatory cytokines and on the clinical characteristics of the donor, including age, doses of inotropic agents administered, comorbidities, functional and structural alterations of the cardiac muscle as assessed by echocardiography.

In consideration of the above, the aims of the present study were to evaluate potential donor-related clinical (age, doses of noradrenaline received), echocardiographic (left ventricular ejection fraction and right ventricular function) and laboratory markers of allograft function and/or postoperative PGD, and to determine the association between these markers and the early outcomes of recipients.

## METHODS

The study was approved by the local Research Ethics Committee (protocol no. ETIC 0517.0.203.000-10), and was performed according to the principles of the Declaration of Helsinki. The aims and objectives of the investigation were explained carefully to all potential participants, or their legally authorized representative where appropriate, who were then invited to sign the document of written informed consent to take part in the study.

### Patients

The prospective study involved a paired population comprising 43 donors and 43 recipients who underwent heart transplantation at the University Hospital, Federal University of Minas Gerais, between January 2012 and November 2013. All donors and recipients included in the study were aged 18 years or more and were pairwise compatible for the transplant procedure. Pairs were excluded from the study either when informed consent could not be obtained from both potential participants or when the donated organ could not be harvested for whatever motive.

### Assessment of Donors and Recipients

Potential heart donors were evaluated with regard to demographic data and clinical and biochemical parameters collected. In addition, various other measurements were performed, including systolic and diastolic arterial pressures (along with the mean of the two values), ventilation parameters [fraction of inspired oxygen (FiO_2_) and oxygen saturation levels], echocardiographic data [left ventricular ejection fraction (VEF) and anatomical, structural and functional information], times of hospitalization and diagnosis of brain death, and doses of noradrenaline administered. Information regarding the usage and doses of noradrenaline was obtained at the time of admission to hospital. In addition, these data, along with those relating to pressure and ventilation, were collected 48 h and 24 h prior to harvesting, at the preoperative stage immediately before transportation to the operating theatre, at the initiation of harvesting and prior to aortic clamping.

A sample (10 mL) of arterial blood for biochemical analysis was taken from each donor around the time of organ harvesting, transferred to a sterile vial containing heparin (Becton & Dickinson, Franklin Lakes, NJ, USA) and centrifuged at 3000 rpm for 10 min. Five 1 mL aliquots of plasma were separated from each sample, labeled with the names of the donor and the recipient, transported in ice and stored in the freezer at -70°C until required for analysis. Frozen samples were subsequently thawed at room temperature and the concentrations of soluble tumor necrosis factor receptors 1 and 2 (sTNFR1 and sTNFR2, respectively), interleukin-6 (IL-6), IL-10 and monocyte chemoattractant protein-1 (MCP1) were measured using sandwich enzyme-linked immunosorbent assay (ELISA) kits (R&D Systems, Minneapolis, MN, USA), while those of C-reactive protein (CRP) and cTnI were determined using VIDAS^®^QCV and Troponin I ultra (TNIU) assay kits (bioMérieux, São Paulo, SP, Brazil), respectively.

Recipients were evaluated with regard to demographic data and clinical and biochemical parameters collected. Parameters relating to the surgical procedure [cardiopulmonary bypass (CPB), aortic clamping, ischemia and operating times), left VEF, right ventricular function, usage of inotrope/vasodilator medication during and after transplantation, occurrence of PGD, and hospitalization and intensive care unit (ICU) times were recorded. Data relating to the usage of inotropes/vasodilators were obtained immediately before induction of anesthesia, 10 min after withdrawal of CPB, immediately before to transfer to ICU, after one, six, 24 and 48 h in ICU and seven days after surgery.

### Data Analysis and Definitions

Clinical and biochemical data pertaining to donors were associated with recipient outcomes assessed as mortality, left/right ventricular dysfunction on echocardiography, requirement of high doses of inotropes and/or circulatory support with intra-aortic balloon pump for maintenance of cardiac output, together with CPB, hospitalization, operating and ICU times. Graft dysfunction was defined as the need for circulatory support (intra-aortic balloon) and/or intravenous administration of high levels of catecholamines (noradrenaline) for withdrawal of CPB, and the presence of moderate or severe postoperative ventricular systolic dysfunction (left or right) on echocardiography.

Noradrenaline doses were defined as low (≤ 0.23 µg/kg/min), moderate (0.24 to 0.46 µg/kg/min) or high (> 0.46 µg/kg/min). The heart transplant team rejected all allografts from donors who had received noradrenaline doses considered excessively high (> 0.69 µg/kg/min) during their permanence in the allocation centers, except in cases where time and clinical conditions allowed restoration of cardiac function. Left ventricular dysfunctions were defined as absent (VEF ≥ 60%), mild (VEF < 60 to > 45%), moderate (VEF ≤ 45 to > 30%) or severe (VEF ≤ 30%). Right ventricular dysfunctions (absent, mild, moderate or severe) were defined by subjective analysis of the echocardiographic data. ICU and hospitalization times of ≤ 5 and ≤ 25 days, respectively, were considered favorable clinical evolutions (suitable outcomes).

### Surgical Procedures

All transplants were performed by the same surgical team using the bicaval anastomosis technique and following identical procedures for the induction and maintenance of anesthesia (as appropriate) and surgery^[4,13]^. Cardioplegia in the donor was performed using 20 mL/kg of Celsior cardioplegic infusion (Genzyme Polyclonals, Champagne au Mont D'Or, France) at 4°C. The excised heart was soaked in 200 mL of cardioplegic solution at 4°C and transferred to a plastic bag, the temperature of which was maintained during transportation by ice contained in two outer plastic bags. During the implant, the heart received intermittent cardioplegia every 30 min with 10 mL/kg of Celsior cardioplegic infusion.

### Statistical Analysis

Descriptive statistics were expressed in terms of mean, standard deviation of the mean, maximum, minimum, median and percentage values. The Student t test, or the non-parametric Mann-Whitney test where appropriate, was employed to compare two independent groups with respect to the variable of interest. Comparison between categorical variables was performed using the χ^2^ test or the Fisher exact test. The significance of differences was established at the 5% probability (*P* <0.05) level. Pearson's correlation coefficient was used to evaluate the relationship between two variables of interest. All analyses were performed using SPSS software version 21.0 (IBM, Armonk, NY, USA).

## RESULTS

### Characteristics of Donors

The majority of donors (74.4%) were males with mean age around 30 years (range 18 - 54 years). Traumatic brain injury (TBI) caused by a traffic accident was the most common cause of death, followed by hemorrhagic stroke, TBI caused by a gunshot wound, and ischemic stroke. Only 7% of donors had a history of alcoholism and 2.3% of tobacco dependence. Additionally, 95% of donors received hormonal therapy including enteral administration of levothyroxine (1.5 µg/kg) and intravenous administration of methylprednisolone (15 mg/kg) every 24 h immediately after diagnosis of brain death (Table 1).

**Table 1:** Characteristics of donors and recipients, and preoperative manometric data of recipients.

Variable	Recipients (N = 43)	Donors (N = 43)
Male [*n* (%)]	24 (55.8)	32 (74.4)
Age (years)	44.8±12.0	30.1±10.4
Weight (kg)	62.3±8.9	69.7±10.7
Height (m)	1.64±0.9	1.7±0.1
Body mass index (kg/m^2^)	22.3±2.7	23.6±2.4
Body surface area (m^2^)	1.7±0.2	1.8±0.2
PVR (Wood units; before/after sodium nitroprusside)	3.2±2.3/2.6±1.3	-
Cardiac output (L/min)	3.1±0.9	-
Cardiac index (L/min/m^2^)	1.8±1.4	-
TPG (mmHg; before/after sodium nitroprusside)	8.2±4.8	-
Eurotransplant heart donor score (mean)	-	13
Hospitalization time (days; before transplant)	22.4±14.4	6.6±4.7
Use of intra-aortic balloon (*n*)	4	-
Cause of encephalic death [*n* (%)]		
Traumatic brain injury (motorcycle/car accident)	-	17 (39.5)
Hemorrhagic stroke	-	13 (30.2)
Traumatic brain injury (firearm projectile)	-	8 (18.6)
Ischemic stroke	-	2 (4.7)
Other	-	3 (7.0)
Alcoholism	-	3 (7.0)
Tobacco addiction	-	1 (2.3)
Hormonal therapy [*n* (%)]		
Levothyroxine	-	41 (95.3)
Methylprednisolone	-	41 (95.3)
Insulin	-	34 (79.1)
Left ventricular ejection fraction (%)	* 65.4±11.9	66.2±6.2
Etiology of cardiomyopathy [n (%)]		
Chagas disease	20 (46.5)	
Ischemia	10 (23.2)	
Idiopathy	7 (16.2)	
Valve diseases	2 (4.6)	
Other	4 (9.2)	

PVR=pulmonary vascular resistance; TPG=transpulmonary pressure gradient

* After transplant

At the initiation of harvesting, 69.8% of donors exhibited mean arterial pressure in the range 60 to 80 mmHg, while 16.3% presented pressure above 80 mmHg. Furthermore, at the start of harvesting, 44.2% of the donors required more than 40% FiO_2_ in the mechanical ventilator to maintain adequate arterial oxygen supply (≥ 90%) (Table 2).

**Table 2:** Clinical characteristics of donors at various stages during transplant procedure.

Variable	*n* (%)
Mean arterial pressure 48 h prior to harvesting (mmHg)	
40-59	5 (11.6)
60-80	31 (72.0)
>80	7 (16.3)
Mean arterial pressure at initiation of harvesting (mmHg)	
40-59	6 (13.9)
60-80	30 (69.8)
>80	7 (16.3)
Mechanical ventilation - FiO_2_ 48 h prior to harvesting (%)	
=40	32 (74.4)
41-60	5 (11.6)
61-80	4 (9.3)
Mechanical ventilation - FiO_2_ at initiation of harvesting (%)	
=40	23 (53.5)
41-60	7 (16.3)
61-80	12 (27.9)
Use of inotropic agents at admission	
Noradrenaline	31 (72.1)
Dobutamine	1 (2.3)
Use of inotrope/vasodilator immediately before harvesting	
Noradrenaline	24 (55.8)
Sodium nitroprusside	2 (4.7)

FiO_2_=fraction of inspired oxygen

**Fig. 1:**
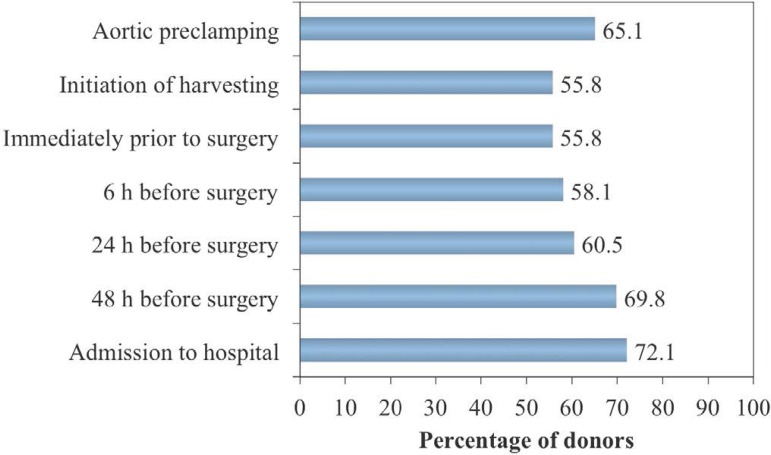
Frequency of donors who received inotropic support (noradrenaline) at different stages during hospitalization.

The majority of donors (89.4%) received noradrenaline at some stage during hospitalization (Figure 1). Relatively low doses of noradrenaline (≤ 0.23 µg/kg/min) were administered during hospitalization to most of the donors (54.3%), while 28.1% received moderate doses (> 0.23 to ≤ 0.46 µg/kg/min), 7% required high doses (> 0.46 µg/kg/min), and 10.6% did not receive noradrenaline. Independent of dose, the highest frequency of administration of noradrenaline (72.1%) was at the time of admission to hospital, while the use of this inotropic agent was much less frequent (55.5%) immediately before surgery, particularly during preparation of the patient for harvesting (administration of hormone therapy and adjustment of clinical management).

The left VEF values of the donors varied between 52% and 80% (mean 66.2±6.2%) with a median value of 65%, and only three (6.9%) donors exhibited left VEF values below the optimum level of ≥ 60%.

### Characteristics of Recipients

Of the 43 recipients, 55.8% were males with mean age around 45 years, mean weight of 62.3 kg and mean height of 1.64 m (Table 1). The pulmonary vascular resistance (PVR) values before and after administration of sodium nitroprusside were 3.2 and 2.6 Wood units, respectively, whereas the mean hemoglobin concentration was 12.5 g/dL at the time of pre-transplant cardiac manometry. The most prevalent etiologies of myocardiopathy among the recipients were Chagas disease (46.5%) followed by ischemia and idiopathic causes. Only one patient underwent retransplant by virtue of cardiac allograft vasculopathy that had evolved over a period of 10 years (Table 1).

The majority of recipients (76.7%) were hospitalized priority patients who had received continuous administration of dobutamine to preserve hemodynamic stability. Ten (39.3%) of these patients (representing of 23.2% of the total number) were in critical state in the ICU and had received high doses of dobutamine or noradrenaline combined with dobutamine to maintain a minimally adequate cardiac output. Despite the gravity of these patients, only three (6.9%) had endotracheal intubation while in the ICU at the time of transplant.

In the post-transplant period, four patients required the insertion of an intra-aortic balloon pump. In one case the device was considered necessary because of the development of PGD with left ventricular dysfunction prevalence, while three patients died due to secondary graft dysfuntion. None of these four patients had isolated right ventricular dysfunction. The left VEF values of the recipients in the first week after transplant varied between 20% and 85% (mean 65.4±11.9%) with a median value of 69%. Only eight recipients (18.6%) presented abnormally low VEF values. The vast majority of recipients had received noradrenaline and/or dobutamine during anesthesia induction and after withdrawal of CPB (Table 3). During transplant, the mean CPB time was 121 min, whereas the mean times of ischemia of the transplanted organ and aortic clamping were 126 and 86 min, respectively.

**Table 3:** Clinical characteristics of recipients at various stages during transplant procedure.

Variable	*n* (%)
Use of inotrope/vasodilator at anesthesia induction	
Noradrenaline	13 (30.2)
Dobutamine	33 (76.7)
Dopamine	1 (2.3)
Adrenaline	1 (2.3)
Sodium nitroprusside	3 (7.0)
Use of inotrope/vasodilator after withdrawal of CPB	
Noradrenaline	15 (34.9)
Dobutamine	42 (97.7)
Adrenaline	3 (7.0)
Sodium nitroprusside	14 (32.6)
Use of inotrope/vasodilator 24 h after surgery	
Noradrenaline	16 (37.2)
Dobutamine	38 (88.4)
Dopamine	1 (2.3)
Sodium nitroprusside	6 (14)
Use of inotrope/vasodilator 1 week after surgery	
Noradrenaline	1 (2.3)
Dobutamine	15 (34.9)
Dopamine	1 (2.3)
Sodium nitroprusside	1 (2.3)
Operating time (h)	5.0±1.3
CPB time (h)	2.0±0.7
Aortic clamping time (h)	1.4±0.3
Ischemia time (h)	2.1±0.5
ICU time (days)	6.9±5.6

CPB=cardiopulmonary bypass; ICU=intensive care unit

### Statistical Analysis of Recipient Outcomes

The recipients with moderate to severe left ventricular dysfunction (VEF ≤ 45%) on echocardiography after transplant had received organs from donors who were significantly older CPB=cardiopulmonary bypass; ICU=intensive care unit (*P* =0.020; Table 4). Furthermore, the recipients of organs from donors who required moderate to high doses of noradrenaline (> 0.23 µg/kg/min) before harvesting exhibited significantly lower VEF values after transplant (*P* =0.002).

**Table 4:** Association between age of donor and right and left ventricular dysfunction in recipient after heart transplant

Outcome of recipient	Age of donor	*P* value
Right ventricular dysfunction		
Absent/mild	29.47 + 10.79 (28.50)	0.498
Moderate/severe	31.55 + 10.21 (33.00)	
Left ventricular dysfunction		
Absent/mild (> 45%)	28.68 + 9.94 (27.50)	0.020 *
Moderate /severe (< 45%)	39.50 + 9.65 (39.00)	
Mortality		
No	29.42 + 10.47 (28.50)	0.151
Yes	36.50 + 8.58 (38.00)	

Data presented as mean + standard deviation (median)

* Mean values significantly different (*P*<0.05) according to Mann-Whitney test.

As shown in Table 5, when plasma levels of sTNFR1 and sTNFR2 in donors were significantly higher (*P* =0.014 and *P* =0.030, respectively), the corresponding recipients remained in ICU for shorter periods (≤ 5 days). Similarly, when IL-6 was significantly higher (*P* =0.029) in donors, the hospitalization times of recipients were shorter (≤ 25 days). Furthermore, when donors exhibited significantly lower plasma concentrations of sTNFR2 and IL-6 (*P* =0.028 and *P* =0.001, respectively), the recipients required moderate/high doses of noradrenaline (> 0.23 µg/kg/min) after being weaned off CPB and during the postoperative period.

**Table 5:** Outcomes of recipients distributed according to the levels of potential cardiac markers in donors.

Potential biomarker in donors	Outcomes of recipients					
	ICU time		Hospitalization time		Moderate/high levels of noradrenaline (> 0.23 µg/kg/min)	
	≤ 5 days (n=24)	> 5 days (n=15)	≤ 25 days (n=32)	> 25 days (n=10)	No (n=27)	Yes (n=16)
	*P*=0.014 *		*P*=0.679		*P*=0.108	
sTNFR2 pg/mL	6218.47+2436.51 (5555.84)	5002.76+2002.69 (4469.48)	5952.36+434.11 (5313.87)	5594.06+2345.63 (4916.84)	6422.48+2342.89 (5537.89)	4913.71+2139.24 (4469.48)
	*P*=0.030 *		*P*=0.545		P=0.028 *	
IL-6 pg/mL	239.84+250.16 (158.10)	117.97+141.61 (87.64)	209.78+205.96 (158.10)	129.61+237.87 (62.53)	254.65+238.06 (164.10)	80.41+80.04 (60.99)
	*P*=0.053		*P*=0.029 *		*P*=0.001 *	
IL-10 pg/mL	243.61+273.32 (133.45)	111.45+173.68 (31.08)	234.50+258.01 (133.45)	64.93+99.15 (14.95)	251.61+267.89 (198.25)	101.27+137.26 (47.42)
	*P*=0.078		*P*=0.037 *		P=0.079	
MCP1 µg/mL	83.37+70.47 (61.06)	64.53+69.43 (35.41)	82.89+71.06 (61.06)	70.73+67.34 (61.35)	91.85+68.56 (64.89)	57.41+65.71 (24.15)
	*P*=0.528		*P*=0.727		*P*=0.092	

sTNFR=soluble tumor necrosis factor receptor; IL=interleukin; MCP=monocyte chemoattractant protein 1; CRP=C-reactive protein; cTnI=cardiac troponin I. Data presented as mean + standard deviation (median).

* Mean values significantly different (*P*<0.05) according to Mann-Whitney test.

Recipients of organs from donors who had received moderate/high doses of noradrenaline (> 0.23 µg/kg/min) during hospitalization remained connected to the CPB pump for significantly longer periods (*P* =0.039) in comparison with those that had received organs from donors not been medicated in this manner (Table 6). Recipients presenting moderate/severe ventricular dysfunctions (left or right) on echocardiography after transplant experienced significantly longer operating and CPB times (*P* =0.038 and *P* =0.022, respectively) compared with those who were not affected by PGD (Table 6).

**Table 6:** Outcomes of recipients distributed according to clinical characteristics of donors and recipients.

Outcomes of recipients	Donors		Recipients	
	Required moderate/high levels of noradrenaline (> 0.23 µg/kg/min)		Postoperative PGD on echo	
	No	Yes	No	Yes
Operating time (h)	4.86 + 1.23 (4.58)	5.38 + 1.32 (5.50)	4.76 + 1.29 (4.00)	5.58 + 1.08 (5.75)
	*P* = 0.228		*P* = 0.038 *	
CPB time (h)	1.87 + 0.53 (1.70)	2.34 + 1.02 (2.09)	1.83 + 0.49 (1.70)	2.49 + 1.02 (2.13)
	*P* = 0.039 *		*P* = 0.022 *	
Aortic clamping time (h)	1.42 + 0.27 (1.28)	1.50 + 0.29 (1.49)	1.39 + 0.27 (1.28)	1.54 + 0.28 (1.58)
	*P* = 0.248		*P* = 0.141	
Ischemia time (h)	2.02 + 0.52 (1.87)	2.24 + 0.46 (2.38)	2.09 + 0.56 (1.96)	2.14 + 0.41 (2.00)
	*P* = 0.067		*P* = 0.512	
ICU time (days)	6.81 + 5.89 (5.00)	7.00 + 5.00 (5.00)	6.25 + 4.91 (5.00)	8.60 + 7.15 (5.50)
	*P* = 0.909		*P* = 0.349	
Hospitalization time (days)	22.15 + 55.85 (15.00)	7.00 + 5.00 (19.50)	20.86 + 11.45 (15.00)	27.70 + 20.80 (19.50)
	*P* = 0.314		*P* = 0.419	

PGD=primary graft dysfunction; CPB=cardiopulmonary bypass; ICU=intensive care unit. Data presented as mean + standard deviation (median)

* Mean values significantly different (*P*<0.05) according to Mann-Whitney test.

Most of the recipients (79.2%) of organs from donors who had not received noradrenaline or who had received at a low dose (< 0.23 µ/kg/min) remained in ICU for a maximum of five days, whereas the majority of patients (66.7%) transplanted with organs from donors who had received moderate/high doses of noradrenaline (> 0.23 µ/kg/min) remained in ICU for more than five days (*P* =0.004; Table 7).

**Table 7:** Outcomes of recipients distributed according to the doses of noradrenaline administered to donors during hospitalization.

Outcomes of recipients	Donors requiring moderate/high levels of noradrenaline (> 0.23 µg/ kg/min)	
	No [*n* (%)]	Yes [*n* (%)]
ICU time (days) a ^,^ c		
≤ 5	19 (79.2)	5 (33.3)
> 5	5 (20.8)	10 (66.7)
	*P*=0.004 *	
Hospitalization time (days) b ^,^ c		
≤ 25	21 (84.0)	8 (57.1)
> 25	4 (16.0)	6 (42.9)
	*P*=0.124	
Right ventricular dysfunction b ^,^ c		
Absent/mild	22 (84.6)	9 (56.2)
Moderate/severe	4 (15.4)	7 (43.8)
	*P*=0.070	
Left ventricular dysfunction b ^,^ c		
Absent/mild	23 (88.5)	12 (80.0)
Moderate/severe	3 (11.5)	3 (20.0)
	*P*=0.651	
Mortality b		
No	25 (92.6)	14 (87.5)
Yes	2 (7.4)	2 (12.5)
	*P*=0.621	

PGD=primary graft dysfunction; ICU=intensive care unit

a Statistical differences determined using the χ2 test.

b Statistical differences determined using the Fisher exact test.

c Patients who died within 48 h after surgery or for whom there was no information available were not included.

* Mean values significantly different (*P*<0.05).

## DISCUSSION

PGD often complicates heart transplantation in the immediate postoperative period, affecting 10% to 40% of allografts depending on the definition adopted, and constitutes the main cause of death. Indeed, PGD is responsible for 40% of deaths within 30 days of transplantation and 18% between 31 days and one year^[14]^. PGD is caused by multiple factors involving problems associated with the heart donor, the organ recipient and surgical management^[15]^. The discovery of donor-related biomarkers that could serve as predictors of allograft quality would facilitate the selection of potential donors and reduce the frequency of postoperative PGD and mortality of recipients.

It has been previously shown that recipients of allografts from older donors (≥ 50 years) exhibit reduced 1 month, 1 year and 5 year survival rates^[16]^. Additionally, Lund et al.^[17]^ have demonstrated that transplants from older donors were associated with progressively reduced survival rates of recipients at 1, 5, 10 and 20 years postoperatively, however, this report did not disclose aspects relating to early morbidity and mortality, particularly during hospital confinement. Nevertheless, donor age is considered a notable predictor of mortality as well as of post-transplant complications, such as cardiac allograft vasculopathy^[16,18]^. It is important to emphasize that the present study focused on donor-related factors influencing allograft function and clinical evolution of patients within the 30 days period after transplantation. As shown in this study, the frequency of moderate/severe left ventricular dysfunction was higher in recipients of organs from older donors, and such circumstances may determine the future survival of these patients.

Intravenous administration of relatively high levels of vasoactive catecholamines, especially noradrenaline, to the donor prior to harvesting or to the recipient during and after transplant has been considered a predictor of PGD^[2,5,12]^. The cut-off point for noradrenaline infusion employed in our study was 0.23 µg/kg/min, and rates above this limit were considered to jeopardize the function of the allograft in the recipient. Earlier reports have described that noradrenaline diffusion rates between 0.06/0.08 and 0.8 µg/kg/min are acceptable^[11,12,19]^. However, it is acknowledged that the inotrope cut-off point represents only one of the clinical parameters of the quality of heart donors to be considered in evaluating the complex transplant process. Administration of higher levels of noradrenaline to donors alone does not contraindicate heart transplantation, since it is not clear that this approach leads to increased mortality despite its association with PGD. The judicious acceptance of the effectiveness of noradrenaline doses > 0.23 µg/kg/min might be fundamental for expanding the number of donor candidates without increasing recipient mortality, as shown by our study in which 37.2% of the donors received moderate/high levels of this medication. Noradrenaline has been elected the vasoactive amine of choice in emergency hospitals and ICUs to treat hypotension refractory to volume in donors, in spite of its well-known deleterious effects on cardiomyocytes and the potential risk of PGD in recipients^[12]^. Our results indicate a significant correlation between administration to donors of noradrenaline doses > 0.23 µg/kg/min and the occurrence of left ventricular dysfunction in recipients, as well as protracted CPB and ICU times. Moreover, our findings suggest a tendency towards increased frequency of right ventricular dysfunction in transplanted patients who received organ from donors treated with moderate/high noradrenaline doses.

Earlier reports have suggested that high levels of the cardiac markers TNF-α, IL-6, cTnT, procalcitonin and BNP are correlated with the administration of high doses of inotropic agents and with some degree of PGD^[2,5-8,12,20]^. However, the results presented herein indicate that higher plasma levels of sTNFR1 and sTNFR2 in donors signal a reduction in ICU time for recipients, while enhanced concentrations of plasma cytokines IL-6 and IL-10 in donors are associated with reduced hospitalization time for recipients. The inflammatory response of donors/recipients can be beneficial to some extent, and a balance between pro- and anti-inflammatory cytokines may be important for the immune system to provide protection against PGD^[21]^. Independent of the mechanism of action (feedback loop, down regulation or cross regulation), the modulator function of the soluble cytokine receptors and/or of pro- and anti-inflammatory proteins are well known^[22,23]^. Moreover, it is possible to hypothesize that the cytokine receptors of the allograft in recipients exhibiting intense inflammatory responses will rapidly reach saturation^[24]^ and, for this reason, such patients tend to be more resistant to post-transplant inflammation and have a more successful postoperative evolution (*i.e.* reduced ICU and hospitalization times).

It is likely that PGD, with its associated high morbidity and mortality, will remain a common complication because of the increasing dependence on marginal donors. It is possible to prevent or minimize PGD by careful matching donors and recipients, and effective management of donor heart preservation. In this context, the search for reliable markers of allograft quality must continue and, according to the evidence gathered so far, a focus on pro- and anti-inflammatory cytokines together with their receptors would appear to represent a promising approach.

### Study Limitations

One of the limitations of this study is that the donor population was pre-selected and all of the organs employed in the transplant procedures were considered to be of good quality. Another limitation was related to the both logistical and legal technical difficulties of obtaining blood samples prior to organ harvesting. In this context, it would have been helpful to analyze blood samples from rejected donors in order to determine if there were differences in the concentrations of cardiac markers. Nevertheless, the results presented herein demonstrated that moderate/high doses of noradrenaline (> 0.23 µg/kg/min) negatively influenced the function of the allograft in the recipient. Furthermore, our study provided extra information about the possible protective roles of pro- and anti-inflammatory cytokines and cytokine receptors (sTNFR1, sTNFR2, IL-6 and IL-10) on the transplanted allograft and the clinical benefits exerted on the recipients.

## CONCLUSION

High levels of sTNFR1, sTNFR2, IL-6 and IL-10 in donors were associated with enhanced evolution in recipients. Allografts from older donors, or from those treated with noradrenaline doses >0.23 µg/kg/min, were more frequently affected by PGD within 30 days of surgery.

**Table:** 

Authors' roles & responsibilities	
RB	Conception and design study; realization of operations and/or trials; data collection; analysis and/or data interpretation; statistical analysis; manuscript redaction or critical review of its content; final manuscript approval
MDS	Analysis and/or data interpretation; manuscript redaction or critical review of its content; final manuscript approval
ALTJ	Conception and design study; analysis and/or data interpretation; final manuscript approval
PHNC	Realization of operations and/or trials; manuscript redaction or critical review of its content; final manuscript approval
MCVM	Manuscript redaction or critical review of its content; final manuscript approval
MAR	Data collection; final manuscript approval
SAA	Data collection; manuscript redaction or critical review of its content; final manuscript approval
CLG	Realization of operations and/or trials; analysis and/ or data interpretation; manuscript redaction or critical review of its content; final manuscript approval
